# Role of Coating Processes on the Corrosion Kinetics and Mechanism of Zinc in Artificial Seawater

**DOI:** 10.3390/ma14237464

**Published:** 2021-12-06

**Authors:** Jitendra Kumar Singh, Soumen Mandal, Raihana Jannat Adnin, Han-Seung Lee, Hyun-Min Yang

**Affiliations:** 1 Innovative Durable Building and Infrastructure Research Center, Hanyang University, 55 Hanyangdaehak-ro, Sangrok-gu, Ansan-si 15588, Korea; jk200386@hanyang.ac.kr; 2 Intelligent Construction Automation Center, Kyungpook National University, 80, Daehak-ro, Buk-gu, Daegu 41566, Korea; sou.chm@gmail.com; 3 Department of Architectural Engineering, Hanyang University, 55 Hanyangdaehak-ro, Sangrok-gu, Ansan-si 15588, Korea; jannat_adnin@yahoo.com

**Keywords:** zinc, coating, corrosion, thermal spray, electrochemical impedance spectroscopy, scanning electron microscope

## Abstract

Zinc (Zn) coating is being used to protect steel structures from corrosion. There are different processes to deposit the coating onto a steel substrate. Therefore, in the present study, a 100 µm thick Zn coating was deposited by arc and plasma arc thermal spray coating processes, and the corrosion resistance performance was evaluated in artificial seawater. Scanning electron microscopy (SEM) results showed that the arc thermal spray coating exhibited splats and inflight particles, whereas plasma arc spraying showed a uniform and dense morphology. When the exposure periods were extended up to 23 d, the corrosion resistance of the arc as well as the plasma arc thermal spray coating increased considerably. This is attributed to the blocking characteristics of the defects by the stable hydrozincite (Zn_5_(OH)_6_(CO_3_)_2_).

## 1. Introduction

The surface of a steel structure must be protected from corrosion due to exposure to open atmosphere or controlled laboratory conditions. This is achieved by the deposition of a metal coating onto the steel substrate. Zinc (Zn) metal is widely used as it acts as a barrier and provides cathodic protection as well [1]. It is used in construction, automobiles, electrical engineering, marine engineering, and petrochemicals as it is anodic in nature and proven to be economical [2,3,4]. Zn is preferred over Al as it is more active in the galvanic series. The protective action of Zn lies in its reaction with atmospheric compounds such as O_2_, H_2_O, and CO_2_, whereby dense, adherent, and insoluble corrosion products that isolate Zn from the atmosphere and act as barriers against the ingress of aggressive ions are formed.

There are different processes for depositing Zn onto a steel substrate, such as hot-dip galvanizing (HDG), electroplating, electrogalvanizing, electrodeposition, ion vapor deposition, and thermal spraying [5,6,7,8,9,10,11], of which HDG is the oldest and most widely used. In this process, the steel surface is first cleaned, pickled with acid and rinsed with water, and a flux is applied to prevent oxidation. It is then immersed in a molten Zn bath at a temperature of 445–455 °C followed by quenching that results in the formation of a coating. A metallurgical interaction occurs between the molten Zn and Fe and forms a metallic bond that acts as a barrier for steel [12]. Electroplating is another alternative method for depositing a Zn coating onto a steel substrate. In this process, a cathode, which is the material to be plated, namely steel and anode (Zn) are required, where the wire connecting the cathode and anode for the current flow is immersed into the electrolyte, allowing the migration of ions from the anode to cathode [5].

In recent years, thermal spray technology has become popular owing to its ease of application and broad range of metals and non-metals that may be used for the deposition of the coatings. The coatings thus deposited are hard, wear-resistant, high-temperature-resistant, tribological, and corrosion-resistant. There are different processes of thermal spraying to deposit the coating, including flame spraying [13,14], electric arc spraying [15,16], high-velocity oxy-fuel coating, and cold spraying [17,18]. The most challenging task is protection against corrosion, which is achieved by the deposition of Zn, Al, or Mg coatings onto the steel substrate [19,20,21,22,23]. Therefore, we have limited the scope of the present study to the deposition of corrosion-resistant metallic coatings onto a steel substrate by the plasma arc and arc thermal spray processes.

Thermal spraying is becoming popular nowadays as it can be used for the on-site repair or refurbishment of steel in construction and infrastructure [24]. In this process, either a metal wire or powder is used as the feed stoke, which is melted and sprayed onto the substrate. However, the arc thermal spray process has the drawbacks of high porosity and poor bonding. These are overcome by the plasma arc spray process in which plasma gas is used to melt the metal, then atomize and propel it onto the substrate to obtain a coating of good quality.

In the present study, a coating of Zn was deposited by arc and plasma arc thermal spray processes onto a steel substrate for protection against corrosion. The effects of the arc and plasma arc thermal spray coating processes on the corrosion mechanism and kinetics of the deposited Zn coating in artificial seawater were studied for different periods of exposure, as stated in ASTM D1141 [25].

## 2. Methods and Materials

### 2.1. Deposition of the Coating

**Figure 1:**
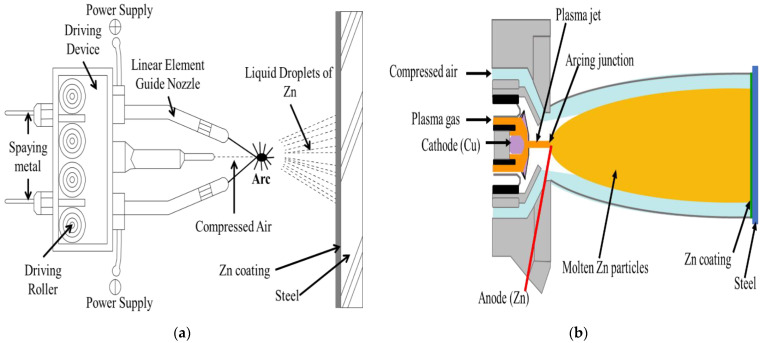
Schematic of (**a**) arc and (**b**) plasma arc thermal spray coating processes.

The Zn coating was deposited by the arc and plasma arc thermal spray processes onto an 8 cm × 6 cm × 0.1 cm steel plate containing C = 0.20, Mn = 0.95, Si = 0.26, P = 0.02, S = 0.01, Cu = 0.02, Cr = 0.04, Ni = 0.03, and Fe = balance in wt %. The steel substrate was polished with 1200 µm emery paper, followed by grit blasting with a 0.8–1.0 mm Al ball to make its surface rough for proper bonding. In both the processes, the coating was deposited by using a Zn (99.95 wt %) wire of 1.6 mm diameter. In the arc thermal spray process, oppositely charged twin wires that protrude with the help of a roller were melted at 30 V and 200 mA at the arcing point. Then, the molten metal particles were propelled through compressed air (7.5 bars) onto the substrate that was kept 15–20 cm away from a spray gun [26,27,28], resulting in the deposition of the coating, as shown in Figure 1a. The plasma arc thermal spray was performed using high-energy plasma gas generated at 55 V, 60 mA, and an air pressure of 3 bars (Table 1). In this process, Cu acted as the cathode (non-consumable) and Zn was the anode (consumable). A single Zn wire was moved to the junction of the arcing point where the cathode was fixed and atomized by the plasma gas. The metal started melting and the molten Zn particles were propelled through compressed air (6 bars) onto the steel substrate that was kept 20–25 cm away from the spray gun, resulting in the deposition of the coating [29], as shown in Figure 1b.

After the deposition of the Zn coating by the different processes, namely arc and plasma arc thermal spray, its thickness was measured at four randomly selected locations using a non-destructive Elcometer 456 gauge (Tokyo, Japan). The average of these measurements was reported to be the coating thickness.

The bond adhesion of four consecutively deposited coatings by the different processes was measured by selecting an area of 16 cm^2^ according to the KS F4716 standard [30], and their average was considered to be the result.

### 2.2. Characterization of the Coatings and Corrosion Products

The cross-section and surface morphology of the coatings deposited by the different processes and the corrosion products formed due to exposure to artificial seawater were characterized by scanning electron microscopy (SEM, MIRA3, TESCAN, Brno, Czech Republic) operated at 15 kV. The elemental analysis was performed using energy-dispersive X-ray spectroscopy (EDS).

The nature of the oxides and corrosion products formed on the deposited coating were determined by X-ray diffraction studies (XRD, Rigaku, Tokyo, Japan) using Cu K_α_ radiation (λ = 1.54059 Å) generated at 40 kV and 100 mA. The probable volume fraction (%) of each phase formed in the corrosion products was calculated using JADE 2016 that was inbuilt in the instrument.

### 2.3. Corrosion Characteristics of the Coatings at Different Exposure Periods

A 1.5 cm × 1.5 cm × 0.1 cm size coupon was cut from an 8 cm × 6 cm × 0.1 cm Zn coating deposited by arc and plasma arc thermal spraying processes for corrosion studies at different periods of exposure to artificial seawater (ASTM D1141) [25]. The solution contained many aggressive ions such as Cl^−^, CO_3_^2−^, SO_4_^2−^, and F^−^, as described in ASTM D1141, that caused the deterioration of the coatings. The pH of the artificial seawater was maintained at 8.2 at 25 (±1) °C by adding 0.1 M NaOH solution. The corrosion resistance of the coating was evaluated using a three-electrode system in which Zn acted as the working electrode (WE), platinum wire as the counter electrode (CE), and Ag/AgCl as the reference electrode (RE). The electrochemical impedance spectroscopy (EIS) measurements were carried out at open circuit potential (OCP) using a VersaSTAT potentiostat (Princeton Applied Research, Oak Ridge, TN, USA) from 100 kHz to 0.01 Hz, with a 10 mV sinusoidal voltage. The potentiodynamic polarization was performed by changing the impressed current from −0.4 V to +0.8 V vs. Ag/AgCl, at a scan rate of 1 mV/s. The analysis of the obtained data was carried out using Metrohm Autolab Nova 1.10. The electrochemical experiments on the coating were performed at 25 (±1) °C in triplicate sets of samples, and their average values were reported.

## 3. Results and Discussion

### 3.1. Characterization of the Coatings

The thickness of the Zn coating deposited by the different processes, namely arc spraying and plasma arc spraying, was measured, and the results are shown in Table 2. The average thickness of arc and plasma arc coatings were 102.25 and 101.75 µm, respectively. The standard deviation was at the range of 1.71–3.50 µm for all coatings. It was considered to be the same for all the processes because it plays a vital role in corrosion resistance. The bond adhesion of the coating was found to be 3.83 (±0.17) and 4.84 (±0.15) MPa for the arc and plasma arc thermal spray processes, respectively. The bonding ability of the Zn coating deposited by the plasma arc thermal spray process was 26.37% greater than that deposited by the arc thermal spray process. This is because in the arc thermal spray process, during the melting of wires at the arcing point, some of the metal particles that did not melt get deposited onto the surface of the substrate, and this causes a reduction in bond adhesion; however, in the plasma arc thermal spray process, Zn melts homogeneously, resulting in higher bond adhesion.

#### 3.1.1. SEM Images of Coatings

**Figure 2:**
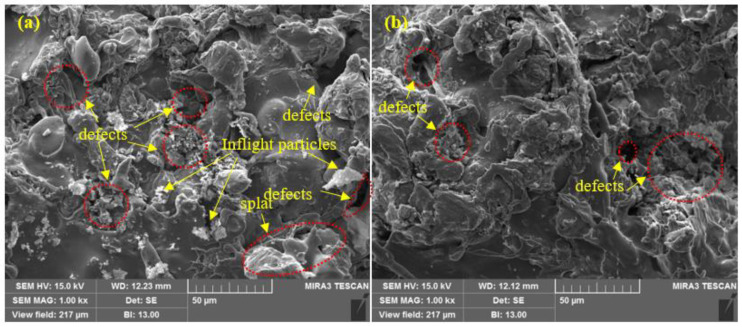
SEM images of the zinc coating deposited by the (**a**) arc thermal spray and (**b**) plasma arc thermal spray processes at 1000×.

**Figure 3:**
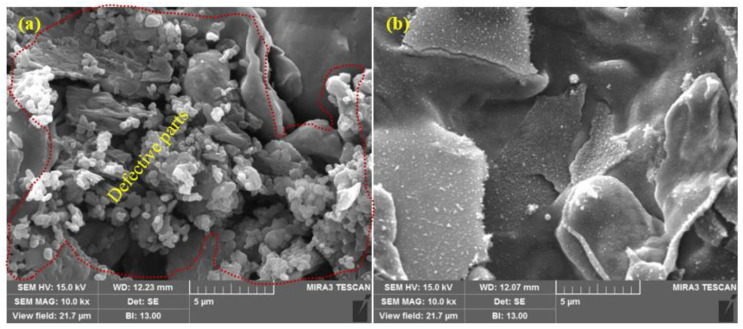
SEM images of the zinc coating deposited by the (**a**) arc thermal spray and (**b**) plasma arc thermal spray processes at 10,000× [31].

**Figure 4:**
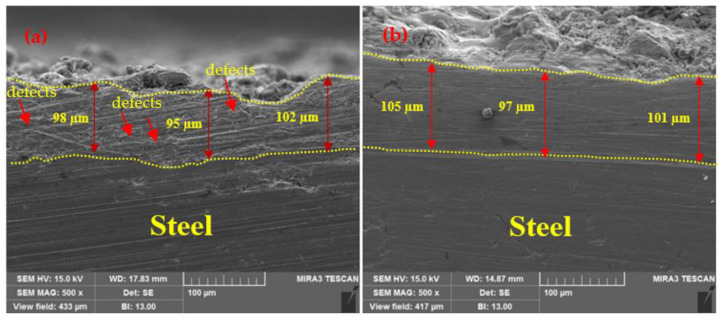
Cross-section SEM images of the zinc coating deposited by the (**a**) arc thermal spray and (**b**) plasma arc thermal spray processes at 500×.

The SEM images of the top surface morphology of the Zn coatings are shown in Figure 2 and Figure 3 [31]. The coating deposited by the arc thermal spray process exhibited severe defects in the form of splats/patches as well as inflight particles, as shown in Figure 2a and Figure 3a. The occurrence of splats is attributed to the sudden cooling of the molten metal droplets that were propelled by the compressed air onto the substrate. In addition, a few smaller molten metal droplets that remained suspended in the air/atmosphere later got deposited as inflight particles [32]. The presence of inflight particles, splats, and un-melted metal particles cause severe defects on the coating surface. These defects are energetically favorable for the segregation of oxygen and other aggressive ions and trigger the dissolution of the coating [33]. It can be seen from Figure 2a and Figure 3a that the defects are of different sizes and orientations. The cross-section SEM image of the arc thermal sprayed Zn coating is shown in Figure 4a, and severe defects can be seen all over the surface as marked by the arrow, as well as some under the coating that correspond with the top surface morphology shown in Figure 2a and Figure 3a. The Zn coating deposited by the plasma arc thermal spray process exhibits an improved morphology, especially a reduction in surface defects (Figure 2b and Figure 3b); however, a few defects are prevalent although the number and size are smaller as compared to those by the arc thermal spray process. This is attributed to the homogeneous melting and sudden cooling of the metal particles that were deposited continuously during spraying. The formation of defects cannot be avoided, but it can be reduced by optimizing the parameters. The kinetic energy of the plasma arc thermal spray is higher than that of the arc thermal spray; hence, the Zn metal melts homogeneously and forms a highly adhesive coating with a dense (Figure 2b) and uniform (Figure 3b) morphology. The cross-sectional SEM image of the plasma arc thermal sprayed Zn coating is uniform and dense (Figure 4b) and in good agreement with the top surface morphology, as shown in Figure 2b and Figure 3b.

The results of the EDS analysis of the deposited coatings are shown in Table 3. An interesting observation that can be seen from this table is that the Zn coating deposited by the arc and plasma arc thermal spray processes exhibited 1.71% and 0.96% of O, respectively. This suggests that during the deposition of the coating, there was no oxidation of Zn, and that this amount of O may have originated from the atmosphere during or after deposition.

#### 3.1.2. XRD Patterns of the Coatings

**Figure 5:**
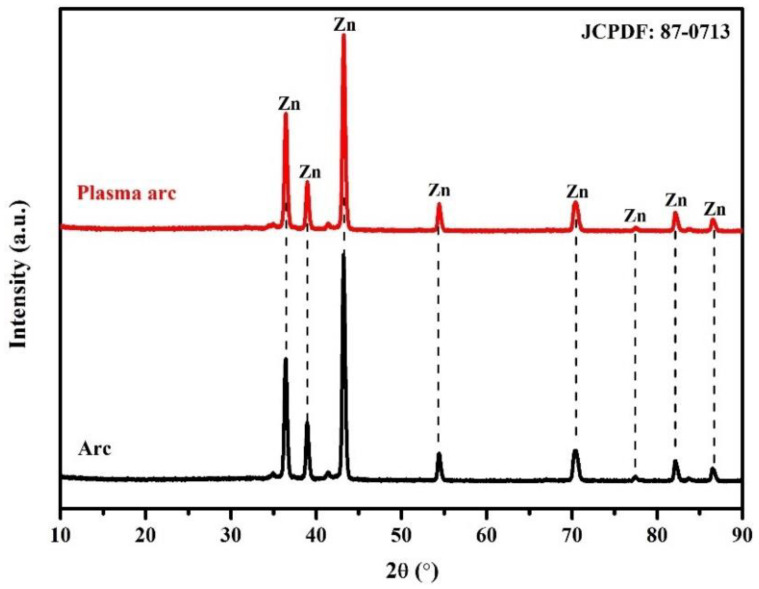
XRD patterns of the Zn coatings deposited by different processes.

The XRD patterns of the Zn coatings deposited by the arc and plasma arc thermal spray processes depicted in Figure 5 exhibited only the presence of Zn (JCPDF: 87-0713), suggesting that there was no oxidation of Zn. This is corroborated by the EDS analysis that showed the amount of O to be 1.71% and 0.96% in the coatings deposited by the arc and plasma arc thermal spray processes, respectively. This amount of oxygen may be enough to oxidize Zn; however, the oxide content was either very low or beyond the detection limit of the XRD instrument.

### 3.2. Corrosion Characteristics of the Zn Coating in Artificial Seawater for Different Exposure Periods

#### 3.2.1. Open Circuit Potential of Coatings

**Figure 6:**
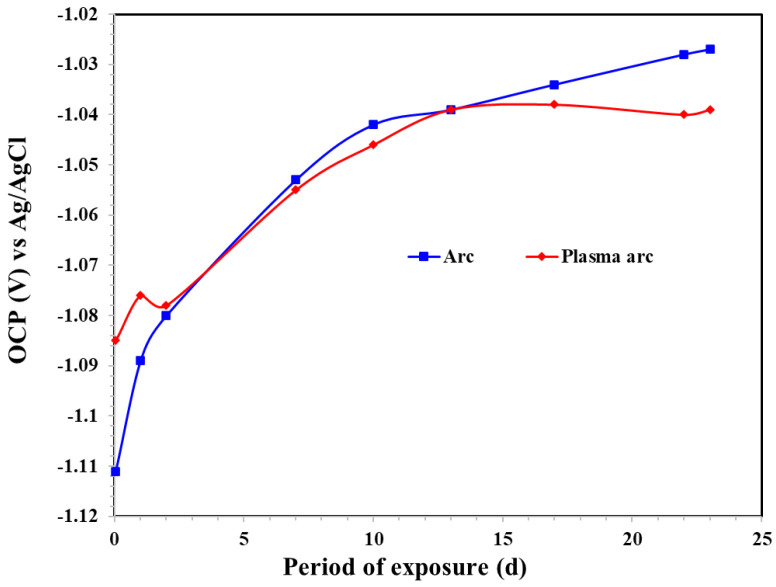
OCP of the Zn coatings in artificial seawater for different exposure periods.

The OCP of the Zn coatings in artificial seawater for different exposure periods is shown in Figure 6. The development of the OCP of the Zn coatings is dependent on the extent of the active area (zinc/steel). The presence of defects enhances the deterioration of the coating as the solution penetrates through them and leads to its dissolution [34]. The Zn coating deposited by the arc thermal spray process initially exhibited an active OCP, from −1.111 to −1.080 V vs. Ag/AgCl from 1 h to 1 d of exposure in artificial seawater, as shown in Figure 6. When the period of exposure was extended, there was a shift in the OCP due to a reduction in the active surface area/site because the defects and surface were filled with corrosion products [32]. The deposition of thick corrosion products such as ZnO/Zn(OH)_2_ onto the top surface led to a reduction of the active surface area of Zn; moreover, the electrical connection between active Zn and the corrosion products stifled the ingress of the solution [35]. The OCP of the Zn coating deposited by the plasma arc thermal spray process was affected by the presence of severe defects/active area on the surface and became a mixed potential owing to the galvanic coupling between the Zn coating and substrate that later enhanced the corrosion. It increased up to 13 d of exposure, after which it became stable. Initially, the coatings deposited by arc and plasma arc thermal spray exhibited defects that enhanced the dissolution of the coating. However, the volume of defects in the plasma arc thermal sprayed coating was lower than that in the arc thermal sprayed coating; more specifically, the arc thermal sprayed coating showed an active OCP up to 1 d of exposure as compared to that of the plasma arc coating. When the exposure period was extended, the corrosion products isolated the active Zn and deposited it onto the coating; the number of defects decreased, making the surface immune to corrosion. Therefore, the Zn coating deposited by the arc thermal spray process required more time for the defects to be filled with the corrosion products; the OCP was not stabilized until 23 d of exposure. The coating deposited by plasma arc spraying required 13 d for the defects to be filled, after which the OCP was stabilized, as seen in Figure 6. The results of the OCP also suggest that the Zn coating deposited by the different processes provided cathodic protection even after 23 d of exposure to the solution.

#### 3.2.2. EIS of Zn Coatings for Different Exposure Periods

**Figure 7:**
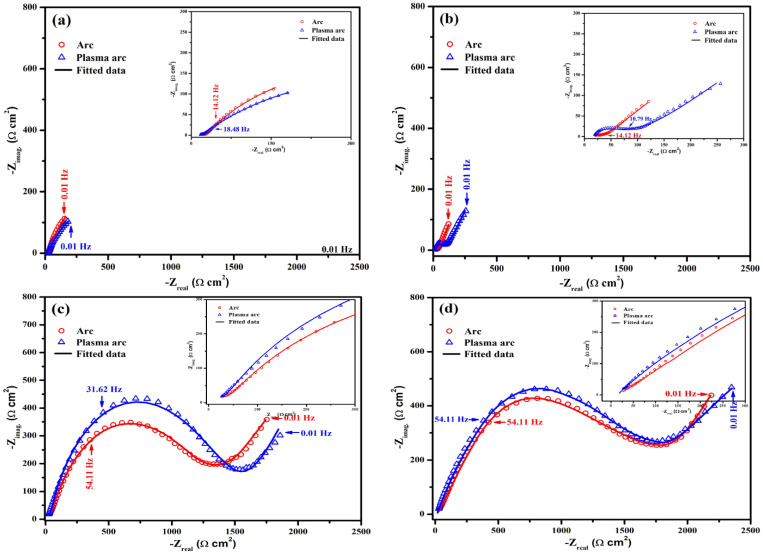
Complex-plane impedance plots of the Zn coatings deposited by different processes after (**a**) 1 h, (**b**) 1 d, (**c**) 13 d, and (**d**) 23 d of exposure to artificial seawater.

**Figure 8:**
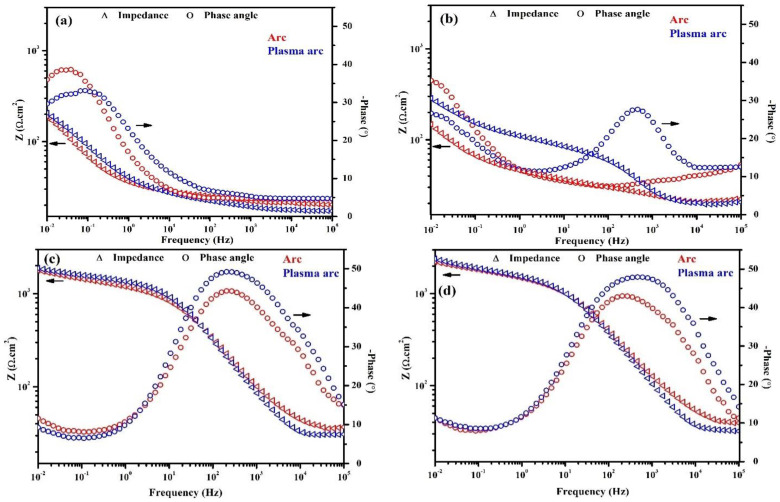
Bode plots of the Zn coatings deposited by different processes after (**a**) 1 h, (**b**) 1 d, (**c**) 13 d, and (**d**) 23 d of exposure to artificial seawater.

The plots of the EIS measurements of the Zn coatings for different periods of exposure to artificial seawater are shown in Figure 7 and Figure 8. The Zn coatings deposited by the arc and plasma arc thermal spray processes exhibited an arc (or depressed semicircle) at high to middle frequency (10^5^–1 Hz), followed by a second ill-defined tail at low frequency after 1 h of exposure (Figure 7a). This is attributed to the charge transfer associated with the effect of ionic double layer capacitance and a finite thickness layer of the diffusion process, which are related mainly to the reduction of oxygen [6]. During a 1 h exposure, the corrosion reaction was initiated, leading to the dissolution of the coating. The arc thermal sprayed Zn coating exhibited severe defects that possessed capacitive properties and enhanced the dissolution of the coating. The defective coating made the surface active owing to the formation of many micro-cells, where the solution penetrated easily and enhanced the dissolution; the lowest total impedance was observed at 0.01 Hz (Figure 8a). Due to its uniformity, the plasma arc thermal sprayed coating exhibited a higher total impedance as compared to that of the arc thermal sprayed coating (Figure 8a). The dissolution of the Zn coatings of the arc and plasma arc thermal spray processes may be caused by the presence of defects.

The penetration of the solution into the substrate requires time and depends on the characteristics of the coating such as morphology, post-treatment, thickness, and surface quality. The morphology of the Zn coating deposited by the arc thermal spray process (Figure 2, Figure 3 and Figure 4) exhibited severe defects, causing an easy penetration of the solution and the occurrence of the oxygen reduction reaction at the coating/steel interface due to galvanic coupling after 1 d of exposure; therefore, it showed a depressed semicircle with a tail at a lower frequency (Figure 7b). This indicates the dominant nature of the cathodic reaction, i.e., the oxygen-reduction reaction, where a sufficient number of electrons following the dissolution of Zn and the diffusion of oxygen through the defects of the coating produce the Warburg impedance. There is a small ill-defined semi-circle from high to middle frequency showing that the weak capacitive properties of charge transfer are attributed to the dissolution of Zn. Thus, the total impedance at 0.01 Hz was the lowest and decreased (Figure 8b) as compared to that for 1 h of exposure and the plasma arc thermal spray coating process. In the present study, the thickness of the Zn coating deposited by the different processes was 100 µm (Table 2). Thus, it required a minimum of 1 d for the solution to penetrate into the coating, whereas Li et al. [36] observed that the penetration of the solution into an 80 µm cold galvanized coating on a steel substrate required 5 h. The present findings suggest that even the coating with the most severe defects, namely the arc thermal sprayed Zn coating, required a minimum of 1 d for the penetration of the solution to cause the oxygen-reduction reaction. The tendency for the dissolution of the Zn coating deposited by the plasma arc thermal spray process is identical to that observed in the arc thermal spray process, but the magnitude of the complex-plane impedance was greater (Figure 7b) than that of the latter after 1 h of exposure. It can be seen from Figure 7b that the magnitude of the capacitive semicircle from high to middle and low frequencies is larger than that for the arc thermal spray process after 1 h of exposure. This result suggests that as the period of exposure was increased, the Zn coating deposited by the plasma arc thermal spray process exhibited more protection against deterioration. Thus, the total impedance at 0.01 Hz was found to be greater than that for the arc thermal spray process and 1h of exposure (Figure 8b). The magnitude of the capacitive loops from high to middle and low frequencies were larger than that for 1 h of exposure. This is attributed to the charge transfer caused by the thin passive/oxide film formed during the initial periods of exposure and the diffusion of oxygen through the defects of the coating that produced the Warburg impedance, as observed in the tail (Figure 7b). The Bode phase angle maxima of plasma arc thermal sprayed coatings (Figure 8b) exhibited a capacitive loop from high to middle frequency at −37° after 1 d of exposure owing to the charge transfer caused by passive film/corrosion products formed during exposure to artificial seawater. The phase angle maxima of the capacitive loop for the plasma arc thermal sprayed coating was higher as compared to the arc thermal sprayed coating because of the formation of corrosion products. In the case of the arc thermal spray, there was no distinct capacitive loop from high to middle frequency, signifying the deterioration of the coating owing to the presence of severe defects. Thus, the lowest total impedance was observed after 1 d of exposure (Figure 8b).

Interestingly, the corrosion resistive properties of the coatings were observed when the period of exposure was extended up to 13 d. It can be seen from Figure 7c that the magnitude of the complex-plane impedance for the coatings of all the processes is higher as compared to that in earlier exposure periods, indicating the corrosion resistive properties of the coating owing to the nucleation and growth of corrosion products that blocked the defects. The diameters of the semicircle from the high to middle and low frequencies were larger as compared to those in earlier exposure periods, suggesting that the corrosion products controlled the corrosion reaction, which enhanced the corrosion properties of the coating. During the initial periods of exposure, the solution penetrated the coating and reacted with active Zn particles (defects), thus forming the corrosion products that produced a barrier; the cathodic reaction took place beneath the coating, making it more compact. During this period of exposure, there was a decline in the cathodic and anodic reactions owing to the formation of corrosion products that blocked the defects/active sites of the coating and reduced the penetration of the solution. Thus, a broad capacitive loop was seen in the high to middle frequency at approximately −50° in the plasma arc thermal sprayed coatings, whereas the arc thermal sprayed coating exhibited a capacitive loop at −43°, as shown in Figure 8c. When the period of exposure was extended, the active Zn particles initially reacted with the solution and deposited the corrosion products, namely ZnO/Zn(OH)_2_, Zn_5_(OH)_8_Cl_2_H_2_O (simonkolleite) and Zn_5_(OH)_6_(CO_3_)_2_ (hydrozincite), onto the surface, which acted as capacitors [37]. Therefore, the total impedance of the Zn coating of the different processes increased after 13 d of exposure as compared to earlier periods, as shown in Figure 7c. The increase in the total impedance was caused because the active area of the Zn coating was reduced, which weakened the electrical connection between the particles and the steel substrate [36]. The corrosion products actively blocked the defects of the coating and resisted the diffusion of the solution [38,39]. Thus, the OCP of the coating increased and then remained stable (Figure 6). This result suggests that at longer durations of exposure, the Zn coating deposited by the different processes provided protection owing to the deposition of corrosion products that dominated over the coating to control the deterioration and block the defects. Moreover, until 13 d of exposure, the tendency for corrosion of the Zn coating was identical for all the processes because its total impedance at 0.01 Hz was almost the same. Initially, the thermal sprayed Zn coatings exhibited defects as the anodic dissolution of the coating was dominant, which allowed the solution to penetrate and initiated the cathodic reaction. It took up to 13 d to deplete the reactive Zn particles and form corrosion products that were deposited onto the coating surface, filling the defects and the region beneath the coating.

As described above, after 13 d of exposure, the tendency for corrosion of the Zn coating was identical for both processes. Thus, it was necessary to evaluate the corrosion resistive properties of the coatings during the extended period, i.e., for 23 d of exposure. The arc and plasma arc thermal sprayed Zn coatings exhibited two large semi-circles, one at high to middle frequency and another at low frequency (Figure 7d), as compared to those in earlier exposure periods. This can be attributed to the formation of stable corrosion products that cause charge transfer, which blocked the defects/active center of the Zn coating and exhibited dielectric properties that reduced the consumption/reaction of Zn [36,40,41,42,43]. This result suggests that there is the possibility of the existence of two layers of corrosion products in the arc and plasma arc sprayed Zn coatings at the coating/solution and coating/substrate interfaces. This was confirmed by the cross-sectional SEM images of the corrosion products and is described in a later section.

**Figure 9:**
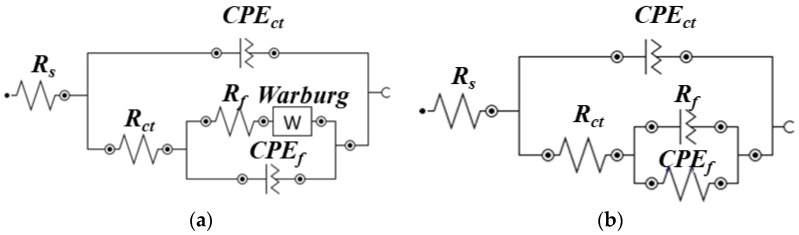
EEC of coatings after (**a**) 1 h and (**b**) 1 d–23 d of exposure to artificial seawater.

The equivalent electrical circuits (EECs) for the fitting of the data obtained by EIS for different periods of exposure in artificial seawater are shown in Figure 9. As explained earlier, the arc and plasma arc thermal sprayed coatings initially exhibited defects; therefore, the EECs of the coatings of both processes after 1 h of exposure to artificial seawater were identical, as shown in Figure 9a. This EEC consists of the resistance of the solution (*R_s_*) in series with two time constants: the first is associated with a constant phase element for charge transfer (*CPE_ct_*) caused by a non-ideal double-layered capacitance at the coating/solution interface, and a charge transfer resistance (*R_ct_*) from high to middle frequency; the second is associated with the anodic dissolution of Zn caused by galvanic coupling with the steel substrate at low frequency with a film/coating resistance (*R_f_*), and a constant phase element for the film/coating (CPE*_f_*) at the substrate/solution interface [44,45,46,47,48,49]. Alternatively, CPE*_ct_* and CPE*_f_* are more relevant than pure capacitors owing to the frequency dispersion caused by the roughness of the corrosion products and the heterogeneity of the coating [41], respectively. When the exposure period is extended from 1 d to 23 d, the Warburg impedance (Figure 9b) appears in series with *R_f_* [50,51].

If the CPE exponent for charge transfer (*n_ct_*) is not equal to 1, then the CPE coefficient (*Q_eff_*) is determined on the basis of an imaginary impedance (*Z_j_*) [52]:

(1) $$Q_{eff}=\sin\left(\frac{nct\pi }{2}\right)\frac{-1}{Z_{j}(f){\left(2\pi f\right)}^{nct}}$$

where *f* is the frequency. If *n_ct_* = 1, then *Q_eff_* becomes a capacitance (*C_dl_*) and Equation (1) can be written as follows:

(2) $$Q_{eff}=C_{dl}=\frac{-1}{Z_{j}(f)(2\pi f)}$$

However, in the present study, *n_ct_* was less than 1 (Table 4) owing to the heterogeneity of the corrosion products. Thus, the blocking characteristics of the coatings owing to the corrosion products between the interfacial capacitance and CPE coefficient (*Q*) can be calculated using Brug’s equation [53] and other equations given in the Ref [54,55]:

(3) $$C_{dl}=Q^{1/n_{ct}}R_{S}^{(1-n_{ct})/n_{ct}{}_{}}$$

The electrochemical parameters after the fitting of the EIS data in the appropriate EEC for different periods of exposure in artificial seawater are shown in Table 4. It can be seen from this table that *R_s_* increases with an increase in the exposure period, which may be attributed to the formation of insoluble oxides/corrosion products that leach out from the surface [56] and reduce the conductivity of the solution. Therefore, the amount of Zn^2+^ leached in the solution can be calculated with the exposure period using Equation (4) [6]:

(4) $$\mathrm{Amount}\;\mathrm{of}\;{\mathrm{Zn}}^{2+}=\frac{MB}{nF}\int^{\;}\frac{dt}{R_{ct}}$$

where M is the atomic weight of Zn, n is the number of electrons exchanged, and F is Faraday = 96,487 C. Table 4 shows the amount of Zn^2+^ ions leached in the solution. It can be seen from this table that for the arc and plasma arc thermal sprayed coatings, 12.10 and 9.11 mg/L Zn^2+^ ions were dissolved in the solution, respectively. When the exposure period was increased, the dissolution of the Zn coating reduced owing to the deposition of insoluble corrosion products in the defects, and the value of *R_ct_* at the coating/solution interface increased. After 1 d of exposure, a negligible amount of Zn^2+^ ions were dissolved in the solution; for larger periods of exposure, there were apparently no Zn^2+^ ions dissolved in the solution, indicating the blocking characteristics of the coating resulting from the corrosion products.

The value of *R_ct_* of the arc thermal sprayed coating decreased for an exposure of 1 d as compared to 1 h, after which it increased, suggesting that initially the coating exhibited defects that rendered the surface active; later, the corrosion products produced a barrier preventing the penetration of the solution. Initially, the arc thermal sprayed coating had severe defects. Thus, the CPE coefficient for charge transfer (*Q_ct_*) increased and *n_ct_* decreased after 1 d of exposure. The defects in the coating or surface enhanced the diffusion of oxygen and caused a cathodic reaction beneath the coating; after 1 d of exposure, all the coatings exhibited Warburg impedance. The arc thermal sprayed coating exhibited the highest Warburg impedance after 1 d of exposure, followed by the plasma arc sprayed coatings. This result suggests that the arc thermal sprayed coating allowed the solution to penetrate and caused corrosion beneath the coating; however, when the exposure period was increased, the Warburg impedance gradually decreased because the corrosion products filled out/blocked the defects in the coating and resisted the ingress of the solution, which diminished the cathodic reaction. Consequently, the plasma arc thermal sprayed coating exhibited the lowest Warburg impedance after 23 d of exposure. In the case of the arc thermal sprayed coating, there was initially a larger active surface area, which led to the formation of a larger amount of corrosion products that blocked the coating, resulting in an increase in *R_ct_* after 23 d of exposure. The plasma arc thermal sprayed Zn coating initially reacted with the solution and formed corrosion products, which blocked the active sites of Zn and diminished the cathodic reaction. Thus, it showed higher values of *R_ct_* and *R_f_* as compared to the arc thermal sprayed coating for all the periods of exposure. An interesting observation can be seen in the *R_f_* values of all the coatings after 13 d of exposure wherein the value increased considerably as compared to that in earlier exposure periods; this indicates that the surface defects were blocked by uniform corrosion products, reducing the ingress of the solution and cathodic reaction. Initially, the defective coating enhanced the corrosion reaction; thus, the *R_f_* value was lower and the values of *Q_f_* and *n_f_* were high. If the coating is defective, the corrosion products also become defective and heterogeneous; thus, the values of *R_ct_* and *n_ct_* were lower, while those of *Q_ct_* and, *C_dl_* were high up to 1 d of exposure in the case of the arc thermal spray process. However, when the exposure period was extended, the corrosion products became uniform and dense, stifling the penetration of the solution and providing barrier protection. Thus, the values of *R_ct_*, *R_f_*, *n_ct_*, and *n_f_* increased, whereas the values of *Q_ct_*, *C_dl_*, and *Q_f_* decreased up to 23 d of exposure for all the coatings. This finding suggests that initially, the corrosion reaction was controlled by the surface morphology, but at longer durations of exposure, it was controlled by the nature and morphology of the corrosion products.

#### 3.2.3. Potentiodynamic Polarization of Zn Coatings after 23 d of Exposure to Artificial Seawater

**Figure 10:**
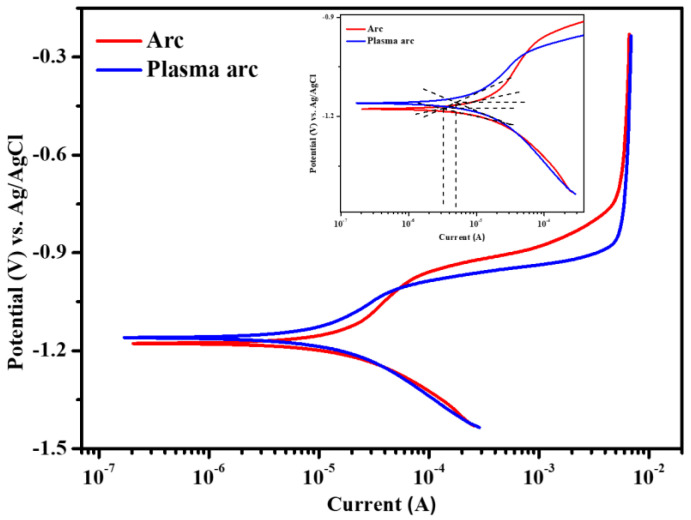
Potentiodynamic polarization plots of the Zn coatings deposited by the arc thermal and plasma arc thermal processes after 23 d of exposure to artificial seawater.

The potentiodynamic polarization of the Zn coating, deposited by the different processes, after 23 d of exposure to artificial seawater is shown in Figure 10. It can be seen from the figure that the coatings deposited by the arc and plasma arc thermal spray processes are cathodically polarized, indicating that the cathodic reaction was controlled by the combined oxygen diffusion-charge transfer process due to the coupling between the coating and the steel substrate. This result is in good agreement with the EIS results, where the diffusion behavior was observed at a low frequency after 23 d of exposure. The cathodic currents of the arc and plasma arc thermal sprayed Zn coatings were almost identical. However, the arc and plasma arc thermal sprayed coatings exhibited a pseudo plateau from −1.120 to −0.960 V vs. Ag/AgCl owing to the charge transfer caused by the corrosion products, and the current was stabilized. The EIS results also confirmed the formation of stable corrosion products from high to middle frequencies, which blocked the defects of the coating. Moreover, a limiting current was observed during the anodic scanning at 4.69 mA, from −0.749 and −0.867 to −0.230 V vs. Ag/AgCl for the arc and plasma arc coatings, respectively. This indicates that the potential of the plasma arc sprayed coating was active at the limiting current and larger, exhibiting the barrier type of protection provided by stable corrosion products [57], which causes mass transfer resistance and blocks the active surface area. The anodic current of the plasma arc thermal sprayed coating was found to be the lowest after *E_corr_*.

The electrochemical parameters were extracted after extrapolating the potentiodynamic polarization plots in the Tafel region. The *E_corr_* of the Zn coating deposited by the arc and plasma arc thermal spraying was found to be −1.175 and −1.157 V vs. Ag/AgCl (Table 5), respectively. This suggests that the coatings of the arc and plasma arc thermal spray processes exhibit more active *E_corr_*, i.e., provide cathodic protection. The plasma arc thermal sprayed Zn coatings exhibited lower corrosion current densities compared to the arc thermal spray process, which is attributed to the deposition of stable, uniform, and thick corrosion products that stifled the ingress of the solution into the steel substrate.

The corrosion rate (C.R.) of the Zn coating deposited by different processes after 23 d of exposure in the artificial seawater solution can be calculated by the following equation [58]:

(5) $$Corrosion\;rate\;(\mu m\;{\mathrm{year}}^{-1})=\frac{3.27\times i_{corr}\times E.W.}{d}$$

where *i_corr_* is the corrosion current density (µA cm^−2^) obtained by dividing the total surface area of the working electrode, i.e., 0.78 cm^2^ in the current. E.W. represents the equivalent weight (g mole^−1^), and *d* is the density (g cm^−3^) of zinc. It can be seen from Table 5 that the plasma arc thermal spray process exhibited the lowest corrosion rate.

### 3.3. Characterization of Corrosion Products

#### 3.3.1. SEM Images of Corrosion Products

**Figure 11:**
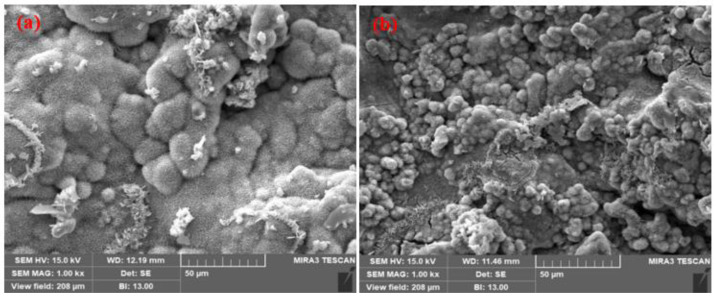
SEM images of corrosion products at 1000× formed on the zinc coatings deposited by the (**a**) arc thermal spray and (**b**) plasma arc thermal spray processes after 23 d of exposure to artificial seawater.

**Figure 12:**
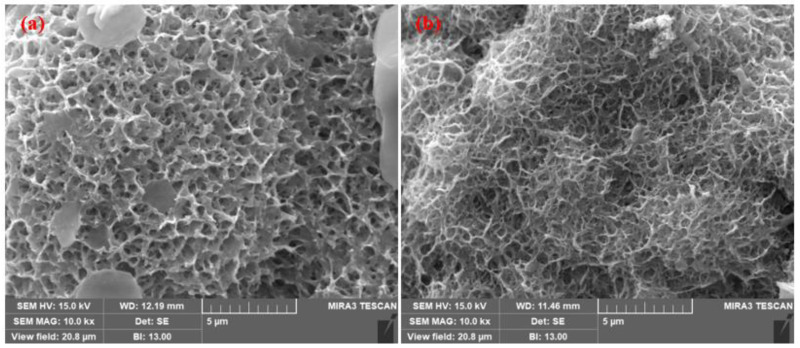
SEM images of corrosion products at 10,000× formed on the zinc coatings deposited by the (**a**) arc thermal spray and (**b**) plasma arc thermal spray processes after 23 d of exposure to artificial seawater.

The SEM images of the corrosion products formed on the Zn coatings after 23 d of exposure to artificial seawater are shown in Figure 11 and Figure 12. It can be seen from Figure 11a,b that the corrosion products formed on the Zn coatings of the arc and plasma arc thermal spray processes, respectively, are sponge-like, globular, dense, and thick, and cover the entire surface uniformly, resulting in improved corrosion resistance. It was reported by Yin et al. (2019) that the sponge and globular types of corrosion products of Zn were mostly composed of hydrozincite (Zn₅(CO₃)₂(OH)₆) [59]. However, in the case of the plasma arc thermal sprayed coating, the corrosion products were globular and smaller in size, making the surface dense and compact (Figure 11b); therefore, the chances of penetration of the solution were negligible.

The SEM images were also taken at a high magnification (10,000×) and the results are shown in Figure 12. Figure 12a shows plate-like corrosion products of the arc thermal sprayed coating embedded in a filamentous, net, and lotus leaf-like morphology, where the pores of the corrosion products opened upwards and exhibited lower corrosion resistance than that of the plasma arc thermal sprayed coating. The plasma arc thermal sprayed coating exhibited dense corrosion products with pores opening upwards (Figure 12b), but under the open pores, a dense network of corrosion products was deposited, which provided protection by blocking the defects of the coating.

**Figure 13:**
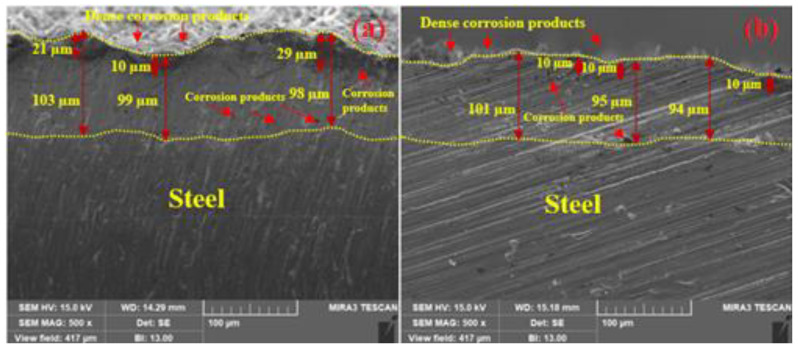
SEM images of cross-sectional views of corrosion products formed on the zinc coatings deposited by the (**a**) arc thermal spray and (**b**) plasma arc thermal spray processes at 500× after 23 d of exposure to artificial seawater.

The SEM images of the cross-sectional views of the corrosion products are shown in Figure 13. Figure 13a shows that the corrosion products formed on the arc thermal sprayed coating are compact and dense with protuberances close to the coating. They were formed due to the ingress of the solution through the defects that reached the substrate and caused galvanic coupling, leading to the cathodic reaction. These corrosion products filled the defects, producing a dense coating; this is the reason why *R_ct_* and *R_f_* increased after 23 d of exposure (Table 4). Moreover, the solution reached a depth of approximately 29 µm, and the color of the coating became gray (Figure 13a) owing to the reaction of Zn with the solution and the formation of corrosion products. This result suggests that corrosion products were formed above the coating as well as under it. The plasma arc thermal sprayed coating showed uniform and dense corrosion products that filled the defects, as shown in Figure 13b. However, the defects were mostly on the top surface, where corrosion products with a thickness of almost 10 µm were deposited; this suggests that the coating consisted of fewer defects, which is consistent with the coating morphology (Figure 2b, Figure 3b and Figure 4b). Moreover, a few tiny defects were connected to the coating; thus, some corrosion products were observed inside the coating that filled the defects and stifled the ingress of the solution, leading to diminished cathodic reaction at longer durations of exposure.

The EDS analysis of the corrosion products after 23 d of exposure is shown in Table 6. The corrosion products contained many elements, such as C, F, Mg, S, K, Ca, Na, and Cl, which originated from the salts of the respective elements present in artificial seawater. The amounts of F, Mg, S, K, and Ca were nominal, but C, Na, Cl, and O were present in high amounts. This suggests that the elements present in minor amounts may have originated from the composition of the solution, whereas those in large amounts were present due to the corrosion products of Zn. Therefore, it is inferred that corrosion products are mostly composed of C, Na, Cl, O, and Zn. Thus, it is important to know their characterization and nature to define the corrosion protection of the coatings deposited by the different processes. The nature of the corrosion products determined by XRD patterns is described below.

#### 3.3.2. XRD of Corrosion Products

**Figure 14:**
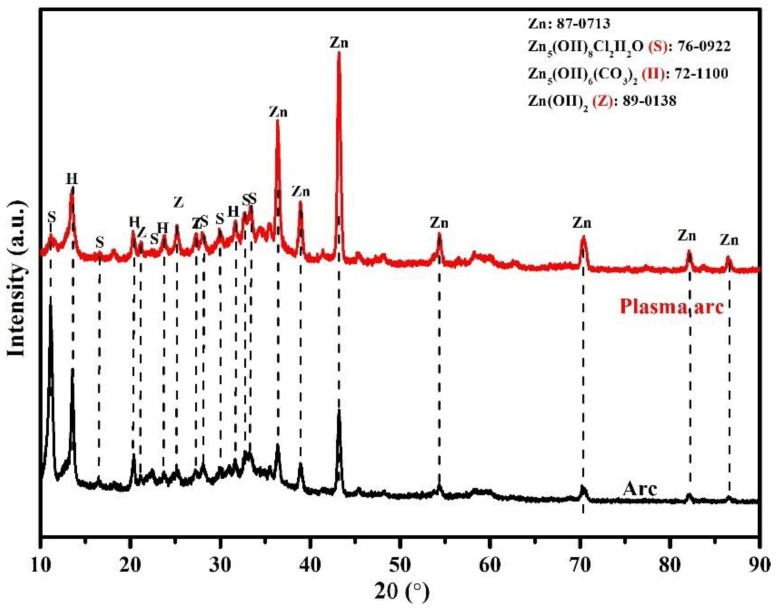
XRD of corrosion products formed on the Zn coatings after 23 d of exposure to artificial seawater.

The nature of the corrosion products after 23 d of exposure to artificial seawater was determined by XRD, and the results are shown in Figure 14. It can be seen that the coatings show Zn (JCPDF:87-0713), Zn_5_(OH)_8_Cl_2_H_2_O (simonkolleite (S): 76-0922), Zn_5_(OH)_6_(CO_3_)_2_ (hydrozincite (H): 72-1100), and Zn(OH)_2_ (zinc hydroxide (Z): 89-0138) as the corrosion products. There is no difference in the phase of the corrosion products because the base metal for corrosion was Zn, and it formed identical phases. However, as the process of deposition of the coating was different, the intensity of the peak ratio was different for the corrosion products. Therefore, it was necessary to quantify each phase and determine the volume fraction (%). This was performed by using the JADE software; the volume fractions of each phase are listed in Table 7. The amount of Zn in the arc thermal sprayed coatings is almost two times lower than that in the plasma arc thermal sprayed coating. This indicates that Zn dissolved and transformed into another form in the arc thermal sprayed coatings, whereas the plasma arc thermal sprayed coating still contained 36% Zn that later provided protection to the steel substrate. The amount of Zn(OH)_2_ in the arc and plasma arc thermal sprayed coatings was almost identical, indicating that this film provided protection during the extended period of exposure. The corrosion products formed on the Zn coating of the arc thermal sprayed process contained higher amounts of simonkolleite as compared to that in the plasma arc thermal sprayed coating. The most interesting observation was regarding the amount of hydrozincite in the coatings. It is thermodynamically highly stable as Zn is situated in both the octahedral as well as the tetrahedral coordination geometry [60] and is sparingly soluble in solution, which signifies the provision of further protection [61]. This means that corrosion at longer durations of exposure was controlled by the presence of hydrozincite. The amount of hydrozincite in the coating of the plasma arc thermal spray process was found to be 33.05%, which was the higher value as compared to the arc thermal sprayed coating. Thus, the lowest corrosion rate was observed in the coating formed by the plasma arc thermal spray process.

## 4. Conclusions

The present study explained the corrosion kinetics and the mechanism of dissolution of the Zn coatings, the nucleation and growth of corrosion products on the coatings, and the blocking characteristics of defects by the corrosion products on the coatings that were deposited by the arc thermal spray and plasma arc thermal spray processes, after being kept in artificial seawater for different periods of exposure. It was concluded that initially, the corrosion phenomena were controlled by the morphology of the coatings, whereas for longer durations of exposure, they were influenced by the nature and morphology of the corrosion products. Initially, the arc thermal sprayed coating exhibited less protection owing to the presence of heavy defects such as splats and inflight particles, which led to galvanic coupling and enhanced the corrosion reaction. The plasma arc thermal sprayed coating showed less defective, compact, and uniform morphology; however, during the deposition process, smaller molten Zn particles adhered to the substrate, making the surface active. Thus, there was no significant improvement in the corrosion resistance of the coating after 1 h of exposure as compared to that of the arc thermal sprayed coating. However, when the period of exposure was extended up to 23 d, the corrosion products blocked the active surface of Zn, resulting in improved corrosion resistance. Moreover, the corrosion products formed on the plasma arc thermal sprayed coatings were dense and compact, producing a higher corrosion resistance with the highest amount of hydrozincite that is thermodynamically stable and sparingly soluble, whereas the arc thermal spray coating process exhibited low corrosion resistance owing to the formation of defective corrosion products.

## Figures and Tables

**Table 1:** Parameters of deposition of the Zn coating using the arc thermal spray and plasma arc thermal spray coating processes.

Parameters	Arc Thermal Spray	Plasma Arc Thermal Spray
Feed stokes	1.6 mm wire	1.6 mm wire
Distance from gun	15–20 cm	20–25 cm
Compressed air pressure (bar)	7.5	1st step: 3 (to generate plasma), 2nd step: 6 (compressed air for spraying)
Spray voltage (V)	30	55
Spraying current (mA)	200	60

**Table 2:** Thickness (µm) of the coatings.

Coatings	Sample Number				Average (µm)	Standard Deviation (µm)
	1	2	3	4		
Arc	98	101	104	106	102.25	3.50
Plasma arc	100	106	102	99	101.75	3.10

**Table 3:** EDS analysis of the Zn coating deposited by different processes.

Coating Process	Element (wt %)	
	O	Zn
Arc	1.71	98.29
Plasma arc	0.96	99.04

**Table 4:** Electrochemical parameters of the Zn coating obtained after the fitting of EIS plots in suitable EECs for different exposure periods in an artificial seawater solution.

Process of Coatings	Period of Exposure	Electrochemical Parameters									Zn^2+^ Amount (mg/L)
		*R_s_* (Ω cm^2^)	*R_ct_* (Ω cm^2^)	*CPE_ct_*		*C_dl_* (µF cm^2^)	*R_f_* (Ω cm^2^)	*CPE_f_*		W (1 × 10^−3^) (Ω cm^2^ s^−0.5^)	
				*Q_ct_* (1 × 10^−5^) (Ω^−1^ cm^−2^ s^−n^)	*n_ct_*			*Q_f_* (1 × 10^−3^) (Ω^−1^ cm^−2^ s^−n^)	*n_f_*		
Arc	1 h	18	102	12.97	0.60	2.28	70	21.57	0.55	-	12.10
Plasma arc		14	115	5.82	0.68	2.05	93	16.0	0.57	-	9.11
Arc	1 d	28	71	20.75	0.55	3.07	56	33.81	0.47	33.81	0.27
Plasma arc		20	149	3.90	0.68	1.34	121	15.58	0.59	13.6	0.16
Arc	13 d	36	315	3.99	0.69	2.11	1410	9.10	0.81	10.57	-
Plasma arc		31	430	2.30	0.71	1.19	1423	9.05	0.81	9.19	-
Arc	23 d	39	455	1.89	0.72	1.14	1738	5.05	0.83	8.25	-
Plasma arc		32	489	1.08	0.73	0.57	1880	5.02	0.84	6.27	-

**Table 5:** Electrochemical parameters obtained after the fitting of potentiodynamic polarization plots in the Tafel slopes.

Coating Process	Electrochemical Parameters		
	*E_corr_* (V) vs. SCE	*i_corr_* (µA cm^−2^)	C.R. (µm year^−1^)
**Arc**	−1.175	5.45	81.71
**Plasma arc**	−1.157	4.90	73.46

**Table 6:** EDS analysis of corrosion products.

Coating Process	Elements (wt %)									
	C	F	Mg	S	K	Ca	Na	Cl	O	Zn
**Arc**	4.93	0.14	0.29	0.82	0.12	0.33	3.63	3.70	18.80	67.24
**Plasma Arc**	5.75	0.11	0.24	0.84	0.14	0.34	2.76	3.67	16.80	69.35

**Table 7:** Volume fraction of each phase found in corrosion products.

Coating Process	Volume Fraction (%)			
	Zn	Zn_5_(OH)_8_Cl_2_H_2_O	Zn_5_(OH)_6_(CO_3_)_2_	Zn(OH)_2_
**Arc**	17.37	43.08	26.80	12.75
**Plasma arc**	36.00	17.43	33.05	13.52

## Data Availability

It is ongoing project work and data cannot be shared.
